# Bacteriocin-Producing *Enterococcus faecium* LCW 44: A High Potential Probiotic Candidate from Raw Camel Milk

**DOI:** 10.3389/fmicb.2017.00865

**Published:** 2017-05-18

**Authors:** Allison Vimont, Benoît Fernandez, Riadh Hammami, Ahlem Ababsa, Hocine Daba, Ismaïl Fliss

**Affiliations:** ^1^Department of Food Science, Faculty of Agriculture and Food Sciences, Institute of Nutrition and Functional Foods, Laval University, Quebec CityQC, Canada; ^2^School of Nutrition Sciences, University of Ottawa, OttawaON, Canada; ^3^Department of Microbiology, Faculty of Natural and Life Sciences, Ferhat Abbas University Sétif 1Sétif, Algeria

**Keywords:** bacteriocin, enterococci, probiotic, intestinal pathogen, listeriosis, cell adhesion

## Abstract

Bacterial isolates from raw camel milk were screened for antibacterial activity using the agar diffusion assay. Ten isolates selected for their inhibition of Gram-positive bacteria were identified by 16S sequencing as *Enterococcus faecium* or *durans*. An isolate named *E. faecium* LCW 44 exhibited the broadest antibacterial spectrum with an inhibitory activity against several Gram-positive strains belonging to the genera *Clostridium*, *Listeria*, *Staphylococcus*, and *Lactobacillus. E. faecium* LCW 44 was shown to produce N-formylated enterocins L50A and L50B, as revealed by mass spectrometry and PCR analyses. This isolate did not harbor any of the virulence factors tested and was shown to be sensitive to all tested antibiotics. It showed high resistance to gastric and intestinal conditions (78 ± 4% survival). Its adhesion index was evaluated at 176 ± 86 and 24 ± 86 on Caco-2 cells and HT-29 cells, respectively, and it significantly reduced adhesion of *Listeria monocytogenes* by 65 and 49%, respectively. In Macfarlane broth (simulating the nutrient content of the colon), counts of *L. monocytogenes* were reduced by 2 log_10_ cycles after 24 h in co-culture with *E. faecium* LCW 44, compared to the increase of 4 log_10_ cycles when cultured alone. Comparison with a bacteriocin-non-producing mutant of *E. faecium* LCW 44 strongly suggests that inhibition of *L. monocytogenes* was due to bacteriocin production. Altogether, *E. faecium* LCW 44 thus has potential for use as a probiotic for humans and veterinary medicine.

## Introduction

Camel milk is produced mainly in sub-Saharan Africa, where it is a staple food for nomadic peoples (Gerosa and Skoet, 2012). The few published studies on its microbiota have shown that, like other milks, camel milk is a source of lactic acid bacteria, primarily lactobacilli, lactococci, and enterococci (Quigley et al., 2013; Kadri et al., 2015). Nevertheless, differences in the composition of camel milk compared to cow milk (Farah, 1993; Ochirkhuyag et al., 1998; Konuspayeva et al., 2007) and the desert environment away from anthropogenic pressure could underlie significant differences in its microbial ecosystem and the biological characteristics thereof. Camel milk is therefore worth studying as a potential source of yet uncharacterized bacteriocin-producing or otherwise biologically active lactic acid bacteria.

Enterococci, in particular *Enterococcus faecalis* and *Enterococcus faecium*, are common in milk (Bhardwaj et al., 2009) and are also ubiquitous in the environment, animal and human gastrointestinal tracts and traditional fermented foods (Moreno et al., 2006). The genus *Enterococcus* includes a wide range of strains, in some cases suitable as starter cultures or probiotics and in other cases known as spoilage or pathogenic microorganisms (Moreno et al., 2006). Some multidrug-resistant strains, in particular vancomycin-resistant enterococci, are significant causes of nosocomial infections (Arias and Murray, 2012). Their ability to survive adverse conditions, including high-temperature and high-salinity environments, and their adaptability to different growth substrates and conditions, may also allow them to cause spoilage of various foods, especially meat (Björkroth et al., 2005). In contrast, some enterococci play a positive role in various fermented foods, including dairy and vegetable products, apparently through proteolysis, lipolysis, exopolysaccharide production and citrate breakdown (Giraffa, 2003; M’hir et al., 2011). Two strains of *Enterococcus* are currently recognized as probiotics and are commercially available, namely *E. faecium* SF68^®^ (NCIMB 10415, Cerbios-Pharma SA, Barbengo, Switzerland) and *E. faecalis* Symbioflor 1 (SymbioPharm, Herborn, Germany). The efficacy of *E. faecium* SF68^®^ for the prevention and treatment of diarrhea in humans and other animals has been proven, as well as the use of *E. faecalis* Symbioflor 1 as an immune regulator in the treatment of recurrent chronic sinusitis or bronchitis (Franz et al., 2011). Therefore, the use of enterococci in foods or as probiotic must be based on case-by-case studies.

Like other lactic acid bacteria, enterococci are known to secrete antimicrobial substances, making them potentially useful for the prevention of bacterial foodborne illness (Franz et al., 2011; Saris, 2014). Many enterococci produce at least one bacteriocin, which is ribosomally synthesized antimicrobial peptide, active against a wide range of foodborne pathogens including *Listeria* spp. (Nes et al., 2014). According to the classification established by Cotter et al. (2005), most enterocins are class II bacteriocins, defined as non-modified and heat stable. This class includes pediocin-like bacteriocins with a strong anti-*Listeria* activity (class IIa), two-peptide bacteriocins (class IIb), circular bacteriocins (class IIc), and non-pediocin unmodified peptides (class IId). The reason why enterococci have developed such an ability to produce antimicrobial peptides remains unknown but it is most likely that bacteriocin production could be a beneficial trait in some environments to increase competitiveness of the producing strain and to establish its niche among other microbial groups in a hostile environment (Dobson et al., 2012). It has also been suggested that production of bacteriocin could contribute to probiotic functionality in three ways, as colonizing peptides which facilitate the competition of the producer strain with the resident microbiota, as killing peptides which directly eliminate pathogens, or as signaling peptides toward other bacteria or the immune system (Dobson et al., 2012). For instance, Corr et al. (2007) demonstrated that mice can be protected against *Listeria monocytogenes* infection by feeding them the bacteriocin-producing strain of *Lactobacillus salivarius* UCC118, an effect not obtained using a stable non-producing mutant strain. More recently, Kommineni et al. (2015) observed that bacteriocin production by intestinal commensal enterococci in mice reduced vancomycin-resistant enterococci to undetectable levels without significant disruption of the indigenous microbiota. A bacteriocin-encoding plasmid (pPD1) allowed a strain of *E. faecalis* to colonize the mouse gut, replace indigenous enterococci and outcompete *E. faecalis* lacking the plasmid (Kommineni et al., 2015). Bacteriocin-producing enterococci thus show promise as probiotics for preventing or treating intestinal infections. The identification of new enterococcal strains with a high probiotic potential must include rigorous evaluation of their behavior, survival and antimicrobial activity under the physiological conditions of the gastrointestinal tract as well as their safety for such use. It should be noted that neither of the commercially sold enterococcal probiotic strains produces any known bacteriocin.

In the present study, we report our characterization of the probiotic properties and safety of a new strain of bacteriocin-producing *Enterococcus* isolated from camel milk.

## Materials and Methods

### Bacterial Strains and Culture Conditions

Samples of raw camel milk collected in the month of March in two different regions of Algeria (M’sila and El Oued) were diluted serially (10-fold) in peptone water, plated on MRS agar and incubated for 48 h at 30°C. A total of 59 colonies were selected randomly, purified and stocked at -80°C in MRS broth/40% glycerol solution (1:1). All bacterial strains were cultured for 24 h at 30°C or 37°C in BD Difco media (BD, Sparks, MD, United States) unless otherwise indicated. *Listeria*, *Staphylococcus*, and *Streptococcus* species were grown in tryptic soy broth (TSB) supplemented with 0.6% yeast extract. *Escherichia*, *Salmonella*, and *Pseudomonas* species were grown in LB broth. *Clostridium* species were grown in RCM medium in an anaerobic chamber (Forma scientific anaerobic system Model 1025). Lactic acid bacteria including *Carnobacterium*, *Enterococcus*, *Lactobacillus*, *Lactococcus*, and *Pediococcus* were grown in MRS.

### Antibacterial Activity Determination

#### Agar Well Diffusion Assay

The antibacterial activity in neutralized cell-free supernatants was screened against 23 indicator strains using the agar well diffusion method described by Fernandez et al. (2013). Briefly, optimal medium containing 7.5 g/L of agar was inoculated at approximately 40°C with 150 μL of an overnight culture of the target strain and poured into a sterile Petri dish. Wells 7 mm in diameter were then cut in the solidified agar and filled with 80 μL of the cell-free supernatant previously neutralized at pH 6.5 with 5 M NaOH solution. Inhibition zone diameters were measured after 18 h of incubation under optimal conditions for the strain.

#### Microtitration Method

Bacteriocin activity was quantified using the microtitration method described by Fernandez et al. (2013). Briefly, twofold serial dilutions (from 125 μL of culture supernatant) in a microtitration plate (96-well microtest, BD Labware, Franklin, NJ, United States) were inoculated with 50 μL of an overnight culture of the target strain diluted to obtain 5 × 10^4^ cfu/well. After 18 h of incubation at the optimal temperature, the OD at 595 nm was recorded using a spectrophotometer (Infinite^®^ F200 PRO, Tecan Inc., Durham, NC, United States). Bacteriocin activity was defined as the highest dilution showing complete inhibition of the strain (OD equal to the uninoculated control) and was calculated as 2*^n^* × 1,000/125 where *n* is the number of inhibited wells and expressed in arbitrary units per milliliter (au/mL).

### Molecular Identification of Bacteriocin-Producing Strains

DNA was extracted from an overnight culture of each strain using the wizard genomic DNA purification kit (Promega, Madison, WI, United States) as described by Fernandez et al. (2015). The 16S ribosomal DNA was amplified by PCR (Eppendorf Mastercycler gradient, Hamburg, Germany) with 27f 5′-AGAGTTTGATCMTGGCTCAG-3′ (Gürtler and Stanisich, 1996) and 1390r 5′-GACGGGCGGTGTGTACAA-3′ (Zheng et al., 1996) primers (Invitrogen, Carlsbad, CA, United States). The reaction volume was 50 μL, composed of 1 × *Taq* buffer (New England Biolabs, Beverly, MA, United States), 0.25 U of *Taq* DNA polymerase (New England Biolabs), 200 nM of each primer, 200 μM of a dNTP mixture (A, T, C, and G, Invitrogen), and 20 ng of bacterial DNA. The thermal cycle program consisted of an initial cycle of 94°C for 5 min for denaturation and polymerase activation, 35 cycles of 94°C for 45 s, 54°C for 45 s, and 68°C for 60 s, and a final extension step of 5 min at 68°C. PCR products were then subjected to gel electrophoresis (100 V, 1 h) on a 1% agarose gel in Tris-acetate-EDTA 1 × buffer (Ambion, Life Technologies Inc., Burlington, ON, Canada) and visualized by gelRed staining (Biotium, Inc., Hayward, CA, United States). Finally, pure PCR products were sequenced using an ABI 3730XL DNA analyzer (Applied Biosystems, Streetsville, ON, Canada).

### Generation of Non-bacteriocin-Producing Mutant *E. faecium* LCW 44d

Bacteriocin-deficient mutants of *Enterococcus faecium* LCW 44 were obtained using the method described previously by Criado et al. (2006). MRS broth containing novobiocin (1 μg/mL, Sigma, Oakville, ON, Canada) was inoculated with 10^5^ cfu/mL and incubated for 72 h at 30°C. The culture was then diluted serially (10-fold) and plated on MRS agar. A total of 55 colonies were selected randomly, grown separately in MRS broth for 18 h at 30°C and the activity in the supernatant was assessed using the agar diffusion method against *Pediococcus acidilactici* UL5 and *Listeria ivanovii* HPB 28. A non-inhibitory variant thus found was named *E. faecium* LCW 44d. The presence and the absence of plasmid in *E. faecium* LCW 44 and LCW 44d, respectively, was confirmed by plasmid profile analysis. Plasmid DNA was isolated using the GeneJET plasmid extraction kit (Thermo Fisher Scientific Inc., Mississauga, ON, Canada) according to the manufacturer’s instructions. Overnight culture (10 mL) was centrifuged at 5,000 × *g* for 10 min and the cell pellet was lysed for 30 min at 37°C with 40 mg/mL of lysozyme (Sigma) and 200 U/mL of mutanolysin (Sigma). Extraction products and the molecular mass ladder (lambda phage DNA digest by HindIII, Ward’s science, VWR Canada, Mississauga, ON, Canada) were heated for 3 min at 65°C, placed on ice and then resolved immediately by electrophoresis on 0.7% agarose gel in Tris-Acetate-EDTA buffer (1×, Ambion) at 7 V/cm and stained with gelRed (Biotium).

### Molecular Identification of Bacteriocins Produced by *E. faecium* LCW 44

#### Bacteriocin Purification and Identification

A chemically defined medium (Zhang et al., 2009) inoculated with *E. faecium* LCW 44 culture at 1% by volume was incubated for 18 h at 30°C. Cell-free supernatant was loaded onto a Sep-Pak C18 cartridge (Waters, Milford, MA, United States) regenerated with 200 mL of methanol and equilibrated with 200 mL of ultrapure water. Adsorbed compounds were eluted with a discontinuous gradient of acetonitrile (0, 25, 50, 75, and 100%) in 5 mM HCl (250 mL per fraction). Acetonitrile was removed from eluted fractions using a rotatory evaporator. The active fraction was loaded onto a preparative C18 column (Luna 10 μm, 250 mm × 21.10 mm, Phenomenex, Torrance, CA, United States) using a Beckman Gold System (Beckman Coulter, Mississauga, ON, Canada). Sample separation was performed at a flow rate of 10 mL/min using a 20–60% linear gradient of acetonitrile in 5 mM HCl and monitoring OD at 214 nm. The most active fraction was concentrated under a continuous flow of nitrogen, injected into a C18 analytical column (Aeris^TM^ 3.6 μm, PEPTIDE XB-C18, 250 mm × 4.6 mm, Phenomenex) and eluted with a linear gradient of 40–50% acetonitrile at a flow rate of 1 mL/min and a gradient of 0.5%/min. Peptides were monitored at 214 nm, collected separately and assessed for antibacterial activity. Active peptides were then analyzed by matrix-assisted laser desorption ionization tandem time-of-flight mass spectrometry (MALDI-TOF MS) on an AB SCIEX 4800 Plus MALDI-TOF/TOF instrument as described previously (Hanchi et al., 2016). The raw centroid data were then searched using the Mascot search engine v2.10 (Matrix Science) against a custom database (*E. faecium*, 267,757 entries). Deamidation of asparagine and glutamine, and oxidation or formylation of methionine were specified in Mascot as variable modifications. Searches were performed with a precursor mass tolerance of 15 ppm and fragment ion tolerances of 0.6 Da.

#### Genetic Detection of Enterocin Genes

*E. faecium* LCW 44 and LCW 44d were screened by PCR for enterocin L50A and L50B genes as described by Hanchi et al. (2014) using enterocin-specific primers that amplified both L50A, L50B, 7A, 7B, and MR10 (Liu et al., 2011). The PCR cycle consisted of an initial denaturation at 94°C for 5 min followed by 35 cycles of 94°C for 1 min, annealing at 55°C for 1 min, elongation at 68°C for 1 min and final extension at 68°C for 5 min. PCR products were analyzed by electrophoresis on a 1.5% agarose gel as described above.

### Safety Evaluation of *E. faecium* LCW 44

#### Antibiotic Susceptibility

The susceptibility of *E. faecium* LCW 44 was assessed according to the Clinical and Laboratory Standards Institute guidelines (CLSI), using seven antibiotics from six different classes, including penicillin G, streptomycin, gentamicin, vancomycin, erythromycin, chloramphenicol, and tetracycline, as described by Hanchi et al. (2014).

#### Presence of Genes Encoding Virulence Factors

*E. faecium* LCW 44 total DNA was tested by PCR for the presence of any of six genes involved in the pathogenicity of enterococci, namely enterolysin A (*EntLysA*, Liu et al., 2011), cell wall adhesin (*efaAfs*, Eaton and Gasson, 2001), enterococcal surface protein involved in immune evasion (*esp*, Eaton and Gasson, 2001), aggregation substance (*asa1*, Vankerckhoven et al., 2004), cytolysin (*cylA*, Vankerckhoven et al., 2004), and gelatinase (*gelE*, Eaton and Gasson, 2001). DNA from *Enterococcus faecalis* ATCC 27275 extracted as described above was used as control. All PCR reactions were performed as described by the authors. The presence of the β-hemolysin (cytolysin) and gelatinase were also determined, respectively, by hemolysis test (Semedo et al., 2003) and plating on Todd-Hewitt agar supplemented with 30 g/L of gelatin (Coque et al., 1995).

### Assessment of the *E. faecium* LCW 44 Resistance to Gastrointestinal Stresses

The TIM-1 system (TNO, Nutrition and Food Research Institute, Zeist, Netherlands) was used to assess the behavior of *E. faecium* LCW 44 under intestinal conditions (pH and enzymes) (Minekus et al., 1995). The model consists of four compartments simulating stomach, duodenum, jejunum, and ileum interconnected in series by computer-controlled peristaltic valve pumps. During digestion, the temperature was kept constantly at 37°C. The pHs in the gastric and small intestine compartments were monitored and controlled, initially at 5.5 in the stomach then gradually decreased to 1⋅8 after 90 min of digestion, at 6.5 in the duodenum, at 6.8 in the jejunum, and at 7.2 in the ileum. Enzymatic secretions consisted of pepsin (Sigma) and lipase in the gastric compartment and trypsin (Sigma), porcine pancreatin (Sigma) and porcine bile extract (Sigma) in the duodenal compartment. Hollow fiber membranes connected to the jejunal and ileal compartments provided dialysis of the contents thereof against small intestinal electrolyte solution. Sample distribution throughout the different compartments of the apparatus was calculated from sensor data and expressed as a function of time by means of computer interface and software. Two independent digestions were performed according the method described previously by Fernandez et al. (2014) with some modifications. Briefly, 300 mL of fermented skim milk was introduced and aliquots of 1 mL were taken in duplicate at 0, 30, 60, 90, and 120 min from the gastric compartment, 30, 60, 90, 120, and 180 min from the duodenal compartment, and 60, 120, 180, 240, and 300 min from the jejunal and ileal compartments for viable count determination. The discharge from the ileum (effluent) and the solution remaining in the duodenal, jejunal, and ileal compartments at the end of the digestion (chyme) were also sampled and taken in account for survival calculation.

### Cell Culture

Caco-2 (ATCC HTB-37) and HT-29 (ATCC HTB-38) cells were cultured in MultiCell media (Wisent, Montreal, QC, Canada) at 37°C under a 5% CO_2_ atmosphere as recommended by DSMZ. Cells were seeded in 24-well plates (Sarstedt, Saint-Léonard, QC, Canada) in medium without antibiotic and used at confluence (>90% estimated by microscopic observation, reached after 4 days) to avoid association to the well.

### Adhesion and Competition Assays on Caco-2 and HT-29 Cells

Adhesion and competition assays were adapted from Moroni et al. (2006). After overnight growth, bacterial cultures were washed twice with sterile PBS (5,000 × *g* for 5 min) and re-suspended at 8 × 10^8^ cfu/mL in cell medium without antibiotic or fetal bovine serum. Confluent cell monolayers were washed twice with sterile PBS and drained prior to contact with bacteria. For adhesion assays, 125 μL of *E. faecium* LCW 44, LCW 44d, *Lactobacillus rhamnosus* GG or *Listeria monocytogenes* LSD 529 suspensions and 125 μL of cell culture medium without antibiotic or fetal bovine serum were added per well to achieve a ratio of bacteria to eukaryotic cells of 100:1. For competition assays, 125 μL of *L. monocytogenes* LSD 529 suspension and 125 μL of one of the other bacterial suspensions were added per well, reaching a ratio between bacteria of 1:1 while maintaining for each bacterium a ratio bacteria to eukaryotic cells of 100:1. Plates were then incubated for 60 min at 37°C under 5% CO_2_ atmosphere, drained and rinsed four times with sterile PBS. Cells with adherent bacteria were harvested with 200 μL of trypsin-EDTA for 10 min at 37°C and 300 μL of cell culture medium were added. Adherent lactic acid bacteria were enumerated on MRS agar incubated aerobically at 30°C for 24 h while *Listeria* was counted on PALCAM agar after 48 h of incubation at 37°C. Results are expressed as the adhesion index, which is the number of bacteria adhering per 100 cells.

### Inhibition of *L. monocytogenes* in Medium Simulating Colonic Nutrients

Macfarlane broth, mimicking the composition of human intestinal contents (Macfarlane et al., 1998), was inoculated at 10^5^ cfu/mL with an overnight culture of *L. monocytogenes* LSD 530. Overnight cultures of *E. faecium* LCW 44 or LCW 44d were centrifuged at 5,000 × *g* for 5 min, re-suspended in 0.1% peptone water to remove residual bacteriocin and added to the Macfarlane broth at 10^7^ cfu/mL. The culture (500 mL) was incubated anaerobically at 37°C for 24 h. Samples were taken every 2 h for the first 12 h and after 24 h. Enterococci and *L. monocytogenes* were enumerated on MRS and PALCAM agar, respectively. Bacteriocin production was followed using the microtitration method described above.

### Statistical Analyses

Adhesion indexes of bacterial strains to intestinal cells were compared among treatments using the one-way analysis of variance (ANOVA) general linear model followed by Tukey’s HSD test. *L. monocytogenes* counts in Macfarlane broth in the absence or presence of *E. faecium* were compared using MANOVA for repeated measures (time as repeated measures variable) followed by Tukey’s HSD test. Statistical significance was declared at *P* < 0.05. All statistical analyses were performed using JMP^®^ software version 11.0 (SAS Institute Inc., Cary, NC, United States).

## Results

### Screening of Strains for Antibacterial Activity

The 59 bacterial colonies isolated from camel milk were screened against *Pediococcus acidilactici* UL 5, *Listeria ivanovii* HPB 28, and *Escherichia coli* ATCC 25922. The 10 most active isolates were identified as *Enterococcus faecium* or *durans* using molecular methods. **Table 1** summarizes their inhibition of various Gram-positive and Gram-negative bacteria. Inhibitory activity against *Listeria* species and/or lactic acid bacteria was variable. *E. faecium* LCW 44 exhibited the broadest spectrum of activity, inhibiting all tested *Listeria* species (one *L. innocua*, one *L. ivanovii*, and seven *L. monocytogenes*), most of the lactic acid bacteria, and to a lesser extent *Staphylococcus aureus* ATCC 6538. This strain identified as *E. faecium* was chosen for further characterization (GenBank accession number KU299788). The strains LCW 2, 3, 4, 5, 6, 8, and 25 had a spectrum characterized by low activity against 8 of 9 tested *Listeria* and against lactic acid bacteria. LCW 7 had the narrowest antibacterial activity spectrum, directed against only *Lactobacillus leichmannii* ATCC 4797 and *P. acidilactici* UL 5. No activity against tested Gram-negative bacteria was observed.

**Table 1:** Antibacterial activity of cell-free culture of enterococci isolated from camel milk, as measured in the standard agar diffusion assay.

Target strain	Source^a^	Producer strain^c^									
		
		LCW2	LCW3	LCW4	LCW5	LCW6	LCW7	LCW8	LCW9	LCW25	LCW44
*Clostridium tyrobutyricum*	ATCC 25755	ND	ND	ND	ND	ND	ND	ND	ND	ND	14.0
*Streptococcus agalactiae*	RBL5^b^	0.0	0.0	0.0	0.0	0.0	0.0	0.0	0.0	0.0	0.0
*Carnobacterium divergens*	M35^b^	14.0	14.0	13.0	13.0	13.0	0.0	13.0	7.0	14.0	14.0
*Enterococcus faecalis*	ATCC 27275	8.0	8.0	8.0	8.0	8.0	0.0	9.0	0.0	8.0	10.0
*Enterococcus durans*	61A^b^	0.0	0.0	0.0	0.0	0.0	0.0	0.0	0.0	0.0	0.0
*Lactobacillus salivarius*	19^b^	0.0	0.0	0.0	0.0	0.0	0.0	0.0	0.0	0.0	0.0
*Lactobacillus leichmannii*	ATCC 4797	21.0	22.0	22.0	10.0	21.0	15.0	22.0	17.0	22.0	21.0
*Lactobacillus johnsonii*	ATCC 11506	12.0	12.5	12.5	12.5	12.5	0.0	12.0	9.0	12.0	13.0
*Lactococcus lactis*	ATCC 11454	12.0	12.0	12.0	12.0	11.0	0.0	12.0	8.0	12.0	13.0
*Pediococcus acidilactici*	UL5^b^	16.0	16.0	16.0	16.0	16.0	10.0	16.0	12.0	15.0	17.0
*Listeria innocua*	HPB 13	9.0	9.0	9.0	9.0	9.0	0.0	9.0	0.0	9.0	12.0
*Listeria ivanovii*	HPB 28	8.0	8.0	8.0	8.0	8.0	0.0	8.0	0.0	8.0	11.0
*Listeria monocytogenes*	ATCC 15313	0.0	0.0	0.0	0.0	0.0	0.0	9.0	0.0	0.0	12.0
*Listeria monocytogenes*	ATCC 19111	8.0	8.0	8.0	8.0	8.0	0.0	9.0	0.0	9.0	11.0
*Listeria monocytogenes*	ATCC 19115	8.0	8.0	8.0	8.0	8.0	0.0	9.0	0.0	9.0	11.0
*Listeria monocytogenes*	LSD 530	10.0	10.0	10.0	10.0	10.0	0.0	10.0	10.0	10.0	13.0
*Listeria monocytogenes*	LSD 532	9.5	9.5	9.5	9.5	9.0	0.0	10.0	0.0	9.5	13.0
*Listeria monocytogenes*	Scott A3^b^	9.0	9.0	9.0	9.0	9.0	0.0	9.5	0.0	9.5	12.0
*Listeria monocytogenes*	LMA 1045	7.0	7.0	7.0	7.0	7.0	0.0	8.0	0.0	8.0	11.0
*Escherichia coli*	ATCC 25922	0.0	0.0	0.0	0.0	0.0	0.0	0.0	0.0	0.0	0.0
*Pseudomonas aeruginosa*	ATCC 15442	0.0	0.0	0.0	0.0	0.0	0.0	0.0	0.0	0.0	0.0
*Salmonella* Enteritidis	MNHN^b^	0.0	0.0	0.0	0.0	0.0	0.0	0.0	0.0	0.0	0.0
*Staphylococcus aureus*	ATCC 6538	0.0	0.0	0.0	0.0	0.0	0.0	0.0	0.0	0.0	8.0


*^a^ATCC, American Type Culture Collection, Rockville, MD, United States; HPB, Health Protection Branch, Health and Welfare, Ottawa, ON, Canada; LSD, Canadian Inspection Agency, Laboratory Services Division, Ottawa, ON, Canada; LMA, Laboratoire de Mycologie Alimentaire, Laval University, Quebec, QC, Canada; LCW, *Enterococcus* isolate. ^b^Our culture collection. ^c^Diameter (mm) of growth inhibition zone (clear). ND, Not determined.*

### *E. faecium* LCW 44 Produces N-Formylated Enterocins L50A and L50B

**FIGURE 1:**
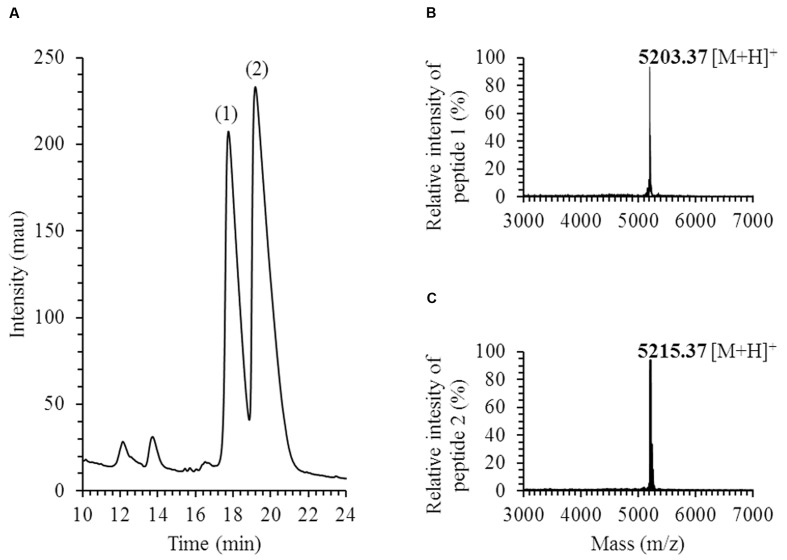
**Purification and identification of enterocins produced by *Enterococcus faecium* LCW 44.** **(A)** HPLC elution profile (chromatogram) at 214 nm; **(B)** MALDI-TOF MS spectrum of enterocin L50B (peptide 1); **(C)** MALDI-TOF MS spectrum of enterocin L50A (peptide 2); mass is mono-isotopic, in Daltons.

Two active peptides, peaks 1 and 2 in **Figure 1A**, were recovered by analytical HPLC in *E. faecium* LCW 44 culture supernatant fractionated by reversed-phase chromatography in three consecutive stages. MALDI-TOF spectra revealed single peaks with mono-isotopic masses of 5,203.37 and 5,215.37 Da, respectively (**Figures 1B,C**). Peptides 1 and 2 were identified by LC-MS/MS, respectively, as formylated enterocin L50B (+28 Da) and formylated enterocin L50A (+28 Da). The experimental b ions closely match those of the theoretically N-formylated forms. Besides, positive amplification with specific primers was observed with *E. faecium* LCW 44 at the expected length (around 275 bp), but no amplification was recorded with the non-producing mutant of *E. faecium* LCW 44d (data not shown). In addition, the presence of a single plasmid (a linear fragment about 9,500 bp in length) in *E. faecium* LCW 44 was confirmed by agarose gel electrophoresis resolution of undigested extracted DNA, and not in *E. faecium* LCW 44d (data not shown).

### Safety Evaluation of *E. faecium* LCW 44

**FIGURE 2:**
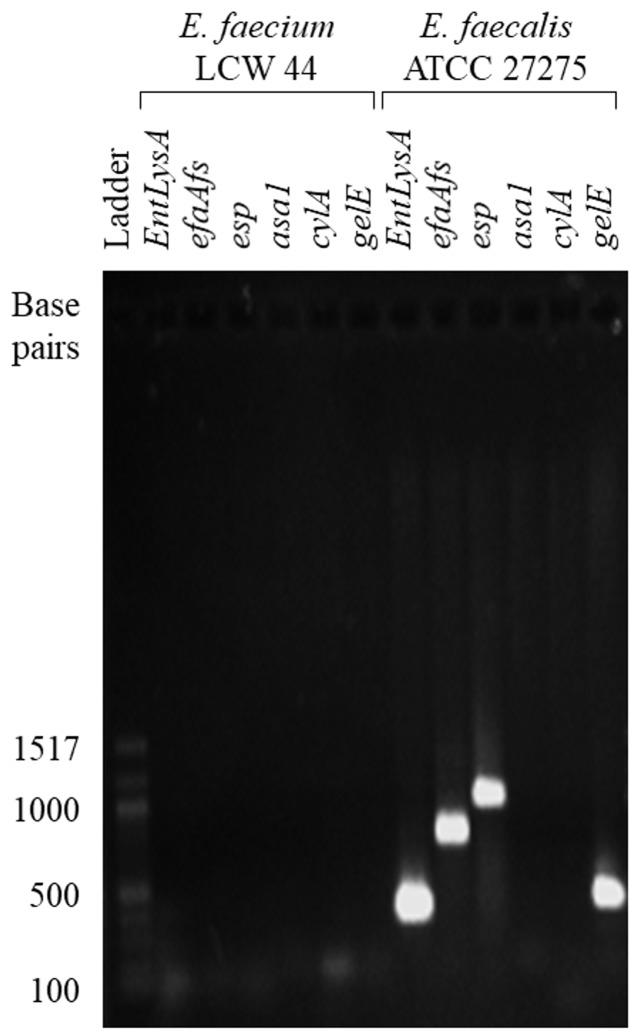
**PCR fragments generated from total genome of *E. faecium* LCW 44 and *E. faecalis* ATCC 27275 using oligonucleotide primers specific for enterolysin A (*EntLysA*), cell wall adhesin (*efaAfs*), surface protein involved in immune evasion (*esp*), aggregation substance (*asa1*), cytolysin (*cylA*) and gelatinase (*gelE*), resolved by electrophoresis on agarose gel**.

*E. faecium* LCW 44 was sensitive to six antibiotics (**Table 2**), namely penicillin G, streptomycin, gentamicin, vancomycin, erythromycin, and tetracycline, and showed intermediate sensitivity to chloramphenicol, with a MIC value of 8.9 μg/mL (breakpoint of 8.0 μg/mL). In addition, none of the virulence genes (*EntLysA*, *efaAfs*, *esp*, *asa1*, *cylA*, and *gelE*) was detected in this isolate (**Figure 2**) and hemolytic and gelatinase activities were absent. In contrast, *Enterococcus faecalis* ATCC 27275 was positive for enterolysin A (*EntLysA*), cell wall adhesin (*efaAfs*), the surface protein involved in immune evasion (*esp*) and gelatinase (*gelE*) as described previously for this strain (McBride et al., 2007), and other *E. faecalis* strains (Hickey et al., 2003; Nueno-Palop and Narbad, 2011). *E. faecalis* ATCC 27275 was negative for aggregation substance (*asa1*), cytolysin (*cylA*), and hemolysis.

**Table 2:** Mean inhibitory concentration of various antibiotics against *Enterococcus faecium* LCW 44.

Antibiotic	Class	Breakpoint^a^ (μg/mL)	MIC (μg/mL)	Interpretation
Penicillin G	β-Lactam	8–16	1.1	Sensitive
Streptomycin	Aminoglycoside	1,000	<27.9	Sensitive
Gentamicin	Aminoglycoside	500	<14.0	Sensitive
Vancomycin	Glycopeptide	4–32	0.6	Sensitive
Erythromycin	Macrolide	0.5–8	0.2	Sensitive
Chloramphenicol	Phenicols	8–32	8.9	Intermediate
Tetracycline	Cyclin	4–16	0.6	Sensitive


*^a^Breakpoints were adopted from CLSI (Clinical and Laboratory Standard Institute).*

### *E. faecium* LCW 44 Was Minimally Affected by Digestive Stresses

**FIGURE 3:**
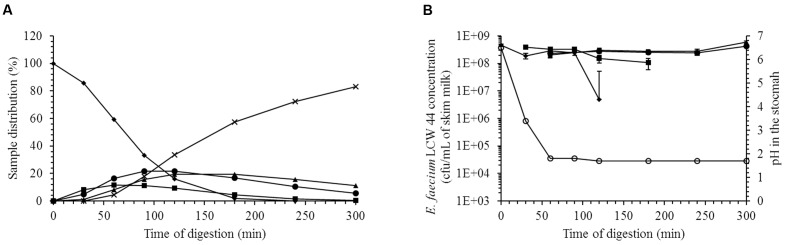
**Viability of *E. faecium* LCW 44 under gastrointestinal conditions (pH and enzymes) in the TIM-1 system.** **(A)** Distribution (%) of the medium (skim milk) in the compartments of the TIM-1 model; **(B)** Viable count (cfu/mL) determined by plating on agar. Stomach (diamond), duodenum (square), jejunum (circle), ileum (triangle), and efflux (cross). All values are the means of two independent experiments. Error bars indicate the standard error of the mean.

The ability of *E. faecium* LCW 44 to survive during passage through the gastric and small intestinal compartments of the TIM-1 model is shown in **Figure 3**. Sample distribution throughout the different compartments of the apparatus is displayed in **Figure 3A**. Starting from an initial concentration of 4.6 × 10^8^ cfu/mL, the survival rate was estimated at 78 ± 4 %. Overall, the different stresses had little impact on the viability of *E. faecium* LCW 44. The most significant decline was observed in the gastric compartment, due most likely to the acidity (pH 1.8).

### *E. faecium* LCW 44 Decreased the Adhesion Rate of *L. monocytogenes*

**FIGURE 4:**
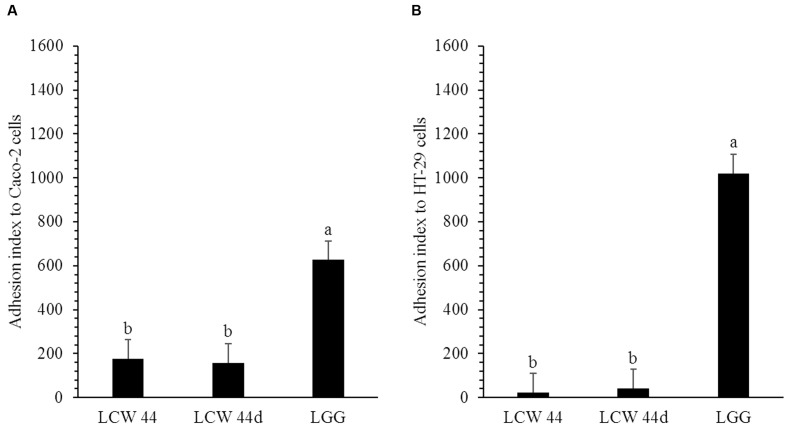
**Adhesion of *E. faecium* LCW 44, LCW 44d and *L. rhamnosus* GG to Caco-2 cells** **(A)** and HT-29 cells **(B)**. Adherent bacteria titer was measured by plating on agar after 1 h of contact. Error bars indicate the standard error of the mean. Different letters indicate significant difference between assays (Tukey’s HSD test, *P* < 0.05, *n* = 3).

**FIGURE 5:**
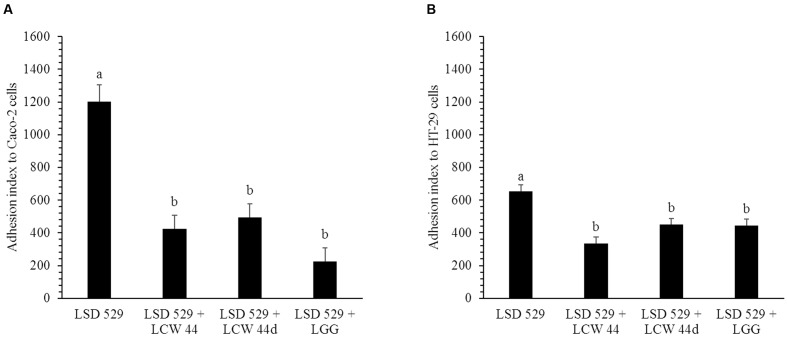
**Adhesion of *L. monocytogenes* LSD 529 to Caco-2 cells** **(A)** and HT-29 cells **(B)** alone and in competition with *E. faecium* LCW 44, LCW 44d or *L. rhamnosus* GG. Adherent bacteria titer was measured by plating on agar after 1 h of contact. Error bars indicate the standard error of the mean. Different letters indicate significant difference between assays (Tukey’s HSD test, *P* < 0.05, *n* = 3).

The adhesion of *E. faecium* LCW 44 and *Lactobacillus rhamnosus* GG to undifferentiated Caco-2 and HT-29 cells is shown in **Figure 4**. No significant difference was observed between *E. faecium* LCW 44 and its non-bacteriocin-producing mutant *E. faecium* LCW 44d, which had adhesion indexes of, respectively, 176 ± 86 and 157 ± 86 on Caco-2 cells and 24 ± 86 and 42 ± 86 on HT-29 cells (*P* > 0.05). These values were significantly lower than those for *L. rhamnosus* GG, 627 ± 86 and 1020 ± 86, respectively, on Caco-2 and HT-29 cells, which is in agreement with previous reports (Jacobsen et al., 1999). Inhibition of *L. monocytogenes* LSD 529 adhesion to intestinal cells by *E. faecium* LCW 44 was evaluated using competition assays (**Figure 5**). Values of 1204 ± 102 and 655 ± 38, respectively, on Caco-2 and HT-29 cells were measured for *L. monocytogenes* alone. However, in the presence of *E. faecium* or *L. rhamnosus* GG, these numbers significantly dropped by 32–81%, regardless of cell line.

### Enterocin Production by *E. faecium* LCW 44 Inhibited *L. monocytogenes* in Medium Simulating Colonic Nutrients

**FIGURE 6:**
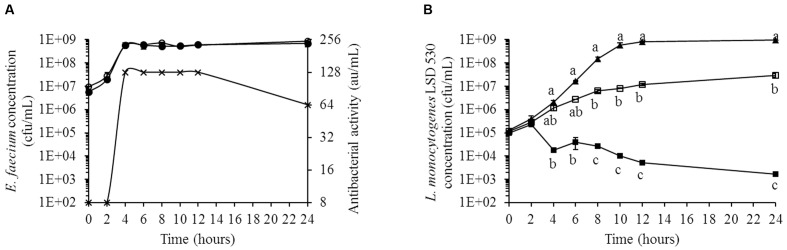
**Growth kinetics of *L. monocytogenes* LSD 530 and *E. faecium* LCW 44 or its non-bacteriocin-producing mutant LCW 44d in Macfarlane medium (simulated intestinal nutrient content).** **(A)** Counts of *E. faecium* LCW 44 (black circle) and *E. faecium* LCW 44d (white circle) in co-culture with *L. monocytogenes* LSD 530, and antibacterial activity (microtitration assay with *P. acidilactici* UL5) of *E. faecium* LCW 44 (cross); **(B)** Growth kinetics of *L. monocytogenes* LSD 530 alone (triangle, black), in co-culture with *E. faecium* LCW 44 (black square) or LCW 44d (white square). Error bars indicate the standard error of the mean. Different letters indicate significant difference between assays (Tukey’s HSD test, *P* < 0.05, *n* = 3).

The inhibition of *L. monocytogenes* LSD 530 by *E. faecium* LCW 44 in Macfarlane broth is shown in **Figure 6**. The variant *E. faecium* LCW 44d (lacking the plasmid on which enterocins L50A and L50B are encoded) was used as control. Although *E. faecium* LCW 44 and its variant LCW 44d showed similar growth kinetics, reaching the stationary phase at around 1 × 10^9^ cfu/mL after 4 h of incubation, only *E. faecium* LCW 44 exhibited significant inhibitory activity against *L. monocytogenes*, reaching a maximum of 128 au/mL at 4 h and remaining constant for at least 8 h before dropping to 64 au/mL at 24 h (**Figure 6A**). Growth kinetics of *L. monocytogenes* cultured alone or in the presence of *E. faecium* LCW 44 or LCW 44d are shown in **Figure 6B**. The pure culture reached the stationary phase after 12 h, with 1 × 10^9^ cfu/mL. In presence of *E. faecium* LCW 44, listerial cell counts were in decline after 4 h and dropped to 2 × 10^3^ cfu/mL after 24 h. In contrast, *E. faecium* LCW 44d was unable to inhibit *L. monocytogenes*, but significantly limited its growth to 3 × 10^7^ cfu/mL after 24 h. Inhibition of the growth of *L. monocytogenes* by *E. faecium* LCW 44 in Macfarlane broth thus may be due to production of enterocins L50A and L50B.

## Discussion

Enterococci are found in diverse ecological niches including dairy products, where they play an acknowledged role in the organoleptic characteristics (Giraffa, 2003). They are also known to produce one or more bacteriocins that inhibit a wide range of foodborne pathogens including *Listeria* spp. (Nes et al., 2014). This feature seems to have an impact on niche competition and to contribute to the control of pathogen infections (Corr et al., 2007; Nes et al., 2014).

In this study, a hitherto unexplored niche was searched for new strains of bacteriocin-producing bacteria. Among 59 isolates from raw camel milk, a few were found to have bacteriocin-like activity. One of these in particular, *Enterococcus faecium* LCW 44, exhibited a broad spectrum of activity against Gram-positive bacteria including *Listeria* spp. This isolate was shown to produce N-formylated forms of enterocins L50A and L50B, two leaderless class IId bacteriocins of 44 and 43 amino acids, respectively. These enterocins are reportedly encoded in the same non-conjugative plasmid in *E. faecium* L50 (Nes et al., 2014) and exhibit an antibacterial activity individually or in combination (Iwatani et al., 2011). Such formylated forms have been identified or suggested in culture supernatant of *E. faecium* isolated from Italian ryegrass (Izquierdo et al., 2008), Spanish dry-fermented sausages (Cintas et al., 1998), and vaginal microbiota in United States (Dezwaan et al., 2007). *Enterococcus durans* 61A, isolated from artisanal fermented milk in Tunisia, has been shown to produce formylated and non-formylated enterocins L50A and L50B, the formylated peptides being six times more abundant (Hanchi et al., 2016).

Though the genus *Enterococcus* includes strains with potential as probiotics or food preservatives (Moreno et al., 2006), it also includes some of the most multi-resistant nosocomial pathogens (Arias and Murray, 2012). *E. faecium* LCW 44 was therefore tested for the factors that contribute most to the emergence of pathogenic strains, namely antibiotic resistance and virulence (Arias and Murray, 2012). It was sensitive to most clinically relevant antibiotics including vancomycin, penicillin G, streptomycin, gentamicin, erythromycin, and tetracycline but showed only moderate susceptibility to chloramphenicol. In comparison, *E. faecium* L50 has been found resistant to clindamycin, nalidixic acid and streptomycin, and moderately susceptible to erythromycin and kanamycin (Basanta et al., 2008). Secreted virulence factors cytolysin, enterolysin A, and gelatinase were not detected in *E. faecium* LCW 44, nor were the cell surface determinants including aggregation substance, cell wall adhesion, and enterococcal surface protein involved in immune evasion. In comparison, *E. faecium* L50 possesses a cell wall adhesin (*efaAfm*) and a sex pheromone (*ccf*) (Basanta et al., 2008). The lack of aggregation protein genes in *E. faecium* LCW 44 corroborates the antibiotic susceptibility since these proteins are encoded in pheromone-responsive plasmids that often also harbor antibiotic resistance genes (Arias and Murray, 2012). The apparent innocuousness of *E. faecium* LCW 44 might be related to its desert origin, far from well-known antibiotic resistance hotspots, namely medical settings and other environments (e.g., intensive livestock production) that are under anthropogenic pressure (Berendonk et al., 2015). Like the probiotic *E. faecium* SF68 (Kayser, 2003), *E. faecium* LCW 44 does not contain the tested virulence genes or the gene for aggregation substance, and thus could be considered as a good candidate for probiotic use in animals or humans. Even though it is expected that conjugation mechanisms would be limited in *E. faecium* LCW 44 by the absence of aggregation substances (Eaton and Gasson, 2001), further investigation should nevertheless be carried out to establish its ability to acquire virulence determinants.

Enterococci are natural inhabitants of the mammalian gut and especially the terminal part of the small intestine (Walter and Ley, 2011; Lebreton et al., 2014). This intestinal segment is also the entry portal of several pathogenic bacteria such as *Salmonella* and *Listeria* (Siebers and Finlay, 1996; Vázquez-Boland et al., 2001). Several groups have examined the possibility of reducing the incidence of these infections using bacteriocin producers such as *Enterococcus mundtii* CRL 35 (Salvucci et al., 2011), *Lactococcus lactis* DPC 6520 (Dobson et al., 2011) or *Pediococcus acidilactici* UL 5 (Dabour et al., 2009). *In situ* production of bacteriocin does indeed appear to provide protection against pathogenic microorganisms (Corr et al., 2007) and to enhance niche competition in the mammalian gastrointestinal tract (Kommineni et al., 2015). In the present study, we evaluated the capacity of *E. faecium* LCW 44 to survive during passage through the stomach and small intestine. A reduction of less than 1 log_10_ cycle was observed, indicating a high capacity for survival under gastrointestinal conditions. Similar resistance has been observed among enterococci including *E. faecium* SF68 (Lewenstein et al., 1979), *Enterococcus faecalis* UGRA10 (Cebrián et al., 2012), and *E. durans* 61A (Hanchi et al., 2014). We evaluated the capacity of *E. faecium* LCW 44 to adhere to intestinal cells but also its capacity to interfere with pathogen adhesion, in particular *Listeria monocytogenes*. These adhesion and competition assays were performed with cells in undifferentiated state since they were reported to be more permissive to this pathogen (Gaillard and Finlay, 1996). The adhesion capacity of *E. faecium* LCW 44 was weak compared to *Lactobacillus rhamnosus* GG, due likely to the absence of aggregation factors that are involved in adhesion mechanisms (Sartingen et al., 2000; Waar et al., 2002). Comparison with literature is difficult due to the discrepancies regarding the methodology and the expression of results. *E. faecium* adhesion nevertheless appeared to approach that of other enterococci. Nueno-Palop and Narbad (2011) reported 2.6 × 10^5^ cfu of *E. faecalis* CP58 adhering to confluent monolayers of Caco-2 cells in 24-well plates, which might correspond to an adhesion index of 26. Sartingen et al. (2000) reported 8.4% of an *E. faecalis* OG1X inoculum adhering to 1–2 × 10^6^ cells, which might correspond to an adhesion index of 420–840. Despite its weak adhesion, *E. faecium* LCW 44 decreased significantly the adhesion of *L. monocytogenes* to both cell lines as much as *L. rhamnosus* GG did. This probiotic is recognized more for its activity against *Salmonella* but has been reported to reduce adhesion of *L. monocytogenes* ATCC 15313 to human intestinal mucus by 53.6 ± 3.6% in a competition assay (Collado et al., 2007). Inhibition of pathogen adhesion by *E. faecium* LCW 44 was weaker than that measured for *E. faecalis* UGRA10, which decreased adhesion of *L. monocytogenes* CECT 4032 to Caco-2 cells by 99.9% (Cebrián et al., 2012). However, it is unclear whether the *E. faecalis* UGRA10 culture was washed before contact with the cell monolayer, and therefore whether enterocin AS-48 produced during overnight growth contributed to inhibiting *Listeria* adhesion. The results of pathogen adhesion/inhibition assays are dependent on the lactic acid bacteria and pathogen tested (Gueimonde et al., 2006) and may involve one or several mechanisms including competition for nutrients, immune stimulation, enhancement of barrier function, competitive exclusion and production of antimicrobial substances (O’Toole and Cooney, 2008). The latter two factors are not likely involved in the effect of *E. faecium* LCW 44, due to its weak adhesion capacity and the fact that the bacteriocin-negative mutant LCW 44d gave similar results.

Finally, *E. faecium* LCW 44 was able to grow and produce its bacteriocins in Macfarlane medium, meaning that the compounds available in the intestine meet its nutritional requirements for growth and production. Bacteriocin production reached a maximum (128 au/mL) in 4 h, in the early stationary phase. Similar results have been reported previously for *L. lactis* UL719 and *P. acidilactici* UL5, which produced their respective bacteriocins in Macfarlane broth after 4 and 6 h, respectively (Fernandez et al., 2013). In the presence of *E. faecium* LCW 44, *L. monocytogenes* counts dropped by 5.8 log_10_ cycles (to 2 × 10^3^ cfu/mL) within 24 h, due likely to production of enterocins L50A and L50B since no such inhibition was not observed in the presence of *E. faecium* LCW 44d. The bacteriocin-negative mutant did nevertheless limit growth of *Listeria* by 2 log_10_ cycles over the same time course. This result is due likely to the greater competitiveness of *E. faecium*, which reached stationary phase twice as fast as *L. monocytogenes. E. faecium* LCW 44 might also produce other antimicrobials active against *Listeria*, since enterococci are known to produce organic acids and multiple bacteriocins (Nes et al., 2007). Inhibition of pathogens by enterococci under intestinal conditions has been reported for *E. faecium* KH 24, which reduced *Salmonella enterica* subsp. *enterica* serovar Enteritidis counts by nearly 1 log cycle in mouse intestines (Bhardwaj et al., 2010). The presence of microbiota induces additional stress that might modulate bacteriocin production. Further studies are needed to confirm that *E. faecium* LCW 44 inhibits *Listeria* in the colon in the presence of intestinal microbiota. We have already observed that *P. acidilactici* UL5 can reduce *Listeria* counts by 2 log_10_ cycles after 6 h in Macfarlane broth (Fernandez et al., 2013) but not in a continuous stirred tank reactor containing immobilized human intestinal microbiota (Fernandez et al., 2015). With its short generation time and high adaptability to different growth conditions, *E. faecium* LCW 44 is expected to compete well with colonic microbiota and to inhibit *Listeria* under gastrointestinal conditions.

## Author Contributions

BF, AV, HD, and IF conceived the research. BF, AV, HD, and AA performed the experiments. BF and AV wrote the manuscript. BF, AV, RH, HD, AA, and IF edited the manuscript. All authors reviewed and accepted the manuscript.

## Conflict of Interest Statement

The authors declare that the research was conducted in the absence of any commercial or financial relationships that could be construed as a potential conflict of interest.
